# The role of HECT-type E3 ubiquitin ligases in DNA damage response and repair

**DOI:** 10.1038/s41420-025-02911-0

**Published:** 2025-12-13

**Authors:** Sara Giovannini, Claudia Fiorilli, Valeria Moriconi, Yufang Shi, Eleonora Candi, Gerry Melino, Francesca Bernassola

**Affiliations:** 1 Department of Experimental Medicine, TOR, University of Rome Tor Vergata, Rome, Italy; 2 The Third Affiliated Hospital of Soochow University, Institutes for Translational Medicine, Soochow University, Suzhou, China; 3 Biochemistry Laboratory, Istituto Dermopatico Immacolata (IDI-IRCCS), Rome, Italy

**Keywords:** DNA damage and repair, Cancer, Ubiquitylation

## Abstract

The post-translational modification ubiquitination consists in a three-step reaction triggered by E1 ubiquitin activating enzymes, E2 ubiquitin conjugating enzymes, and E3 ubiquitin ligases. The latter enzymes, providing substrate specificity, play an important role in determining the fate of the substrate proteins, by regulating their level and function. Efficient DNA damage response (DDR) is necessary to detect and signal DNA damage, thus favoring DNA damage repair to prevent genomic instability and tumorigenesis. Differently from RING (really interesting new gene)-type E3s, the ones belonging to the Homologous to E6AP C-terminus (HECT) family have an intrinsic catalytic activity, which enables them to directly transfer ubiquitin molecules to their substrates. They participate in the regulation of numerous processes, from cell proliferation to apoptosis. Nevertheless, their role in DDR and repair is less known. Recent evidence reports of the HECT E3s involvement in the regulation of DNA damage signaling, chromatin remodeling, repair pathway choice and DNA damage resolution. Further elucidating their functions in DDR and repair may provide new insights into the processes aimed at the preservation of genome integrity, putatively uncovering HECT E3s as therapeutic targets in tumors and defective DNA repair pathologies.

## Facts

- Counteracting DNA damage, DNA damage response and repair mechanisms play a crucial role in preserving genome integrity.
- Ubiquitination regulates the protein level and function of substrates, involved in multiple biological processes, thereby significantly affecting DNA damage repair.
- While the contribution of RING-type E3s to DNA damage repair signaling pathways is extensively reported, the role of HECT-type E3s in the regulation of DNA damage cell response remains less studied.
- Recent evidence unveils a role for HECT E3s in protecting from genome instability and suggests them as putative targets for new cancer therapies.

## Open questions

- Which substrates are modified by HECT E3s in the regulation of DNA damage response and repair?
- How do HECT E3s cooperate with each other, or with members of other E3 families to maintain genome stability?
- Will future screenings and structural studies help in designing more selective inhibitors, able to overcome chemotherapy resistance and reduce off-target effects?

## Background

Ubiquitination regulates cellular processes like cell cycle progression, cell death and DDR [1, 2]. It occurs through a cascade of enzymatic reactions: ubiquitin-activation by E1 enzymes, ubiquitin-conjugation by E2 enzymes and ubiquitin ligation by E3 ligases [3] (Box 1). E3s confer substrate specificity and facilitate regulation and the attachment of ubiquitin molecules to target proteins [4]. The HECT family E3 members are characterized by a unique catalytic mechanism. Unlike other E3s acting as scaffolds, HECT E3s form a thioester intermediate with ubiquitin before its direct transfer to the substrate protein. HECT E3s are involved in many cellular functions, including regulation of cell cycle, stress response, and several modalities of cell death, including apoptosis [5–7], autophagy [8–10], and ferroptosis [11–13], thus influencing tumorigenesis [8, 14–17].

Evidence suggests an important contribution of HECT-type E3s in DDR and repair. Upon DNA damage, cells activate DDR pathways to detect lesions, signal their presence, and coordinate repair mechanisms [18]. Importantly, ubiquitination influences the protein stability and function of various proteins involved in DDR. By controlling their ubiquitination status, HECT E3s can then influence the choice of the repair pathway, the efficiency of repair, as well as the overall response to DNA damage, to finally prevent mutagenesis. Consequently, dysregulation of HECT E3s could lead to defective DNA repair, accumulation of mutations, cancer and even increased resistance to therapies [15, 16].

Compared to other E3 families, like RING E3s, the role of HECT E3s in DDR and repair is less characterized. A better understanding of the mechanisms ensuring genome integrity, by further investigations, may uncover new therapeutic approaches to possibly enhance DNA repair or to sensitize resistant tumors to treatments.

BOX 1 The multiple roles of ubiquitination Ubiquitination is a post-translational modification consisting in the attachment of ubiquitin molecule to specific lysine residues of protein substrates [3]. This mechanism is involved in a variety of cellular processes, such as protein degradation, signaling and DNA repair[77, 151, 204].

**Figure:**
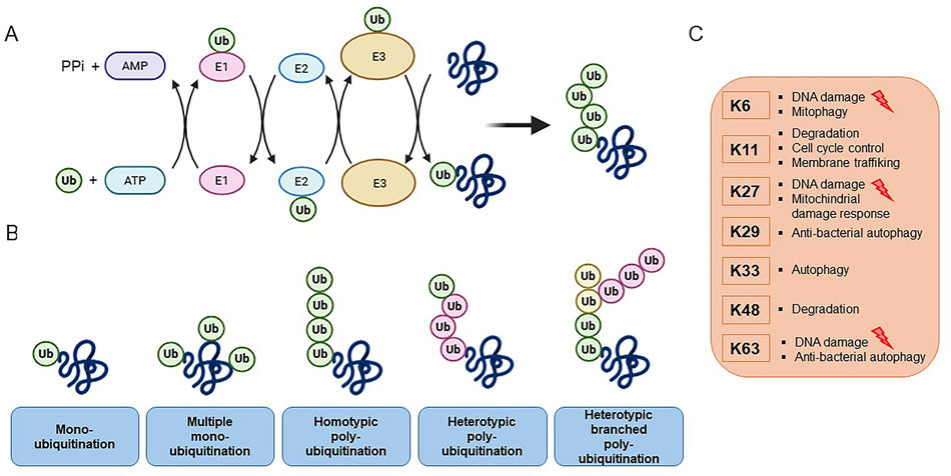
**Box 1 Figure. Different types of ubiquitination and the specific roles of ubiquitin chain linkages.** (A) The key enzymes involved in the ubiquitination cycle: E1, E2 and E3. The process begins with the activation of ubiquitin and its covalent attachment to the E1. In the second step, ubiquitin is transferred to the E2 during conjugation. Finally, the poly-ubiquitin chain is transferred to the target protein through the action of the E3. (B) Formation of different types of ubiquitin chains, including mono-ubiquitination, poly-ubiquitination via the same or different ubiquitin acceptor sites and the formation of branched poly-ubiquitin chains. (C) The different lysine residues involved in the formation of ubiquitin chains determine the fate of poly-ubiquitinated proteins, including their role in DDR.

As shown in the figure, a ubiquitin-activating E1 enzyme starts the reaction by the ATP-dependent formation of a thioester bond between its active cysteine and the ubiquitin molecule. The ubiquitin is afterwards transferred to a conjugating E2 enzyme prior to the recruitment of the ubiquitin-loaded E2 to a specific substrate by a E3 ligase (Box 1 figure). The precise regulation of this mechanism is triggered by this one last enzyme, which recognizes specific structural features of the substrate, to finally transfer a ubiquitin molecule to a particular lysine residue.

This reaction consists of a mono-ubiquitination, whereas poly-ubiquitination occurs when multiple ubiquitin molecules are subsequently attached. As in the figure, poly-ubiquitin chains can be assembled in various topologies, which finally influence the fate and function of the substrate (Box 1 figure) [14, 15]. To form poly-ubiquitin chains, ubiquitin molecules are linked through various lysine (K) residues (K6, K11, K27, K29, K33, K48, K63) [112], to finally determining a specific fate for the substrate, such as proteasomal degradation, functional alterations, subcellular localization, or formation of protein complexes.

Interestingly, specific linkages are associated with particular functions. For instance, K6-linked chains are involved in mitochondrial autophagy (mitophagy) and DDR [204], K27-linked chains, participate in the recruitment of DDR factors and in the modulation of immune responses [205]. On the other hand, K48-linked as well as K11-linked chains are common signals for proteasomal degradation, marking substrates for the 26S proteasome. Differently, K63-linked chains are often involved in non-proteolytic roles such as DNA repair and signal transduction. The structural differences among these poly-ubiquitin chains then influence the activity, localization, or interaction properties of substrate proteins, thus regulating signaling pathways also involved in cell cycle progression, apoptosis, DDR, and repair [15, 206].

Ubiquitination is evidently critical to regulate the level and the function of proteins. The diversity of the ubiquitin linkages and the substrate specificity provided by the E3s affect a variety of processes. Depending on the function of the ubiquitination target and the effects of the modification, E3s are indeed involved in DDR, cell cycle regulation, and cell death [15, 16, 207]. Their ability to modulate the stability of key DDR factors, influencing chromatin remodeling at sites of damage, or facilitating the recruitment of repair factors, underlines the importance of the E3s in influencing cancer and therapy response, thereby contributing to protect genome integrity [15, 16, 97, 102, 207].

## An overview of DDR and repair mechanisms

In response to the presence of DNA damage, signaling pathways, collectively known as DDR, are activated to maintain genome integrity [19, 20]. These signaling cascades trigger cell cycle checkpoints, induce cell cycle arrest and initiate transcriptional events that promote the expression or regulation of DNA repair factors [18, 21]. Defined as the guardian of the genome [22], the transcription factor p53 is the main player of DDR [23–25], as well as other related mechanisms [26–29]. It is involved in cell cycle arrest and activation of transcriptional, repair and apoptotic events, therefore fining controlled in protein level, via an autoregulatory negative feedback loop. Its degradation depends on ubiquitination catalyzed by the RING E3 MDM2 (Mouse double minute 2), one of its principal transcription targets [30]. Depending on the type of DNA damage, DDR signaling induces specific repair mechanisms, such as the double-strand breaks (DSBs) repair pathways, homologous recombination (HR) and non-homologous end joining (NHEJ), as well as the single-stranded DNA repair pathways, nucleotide excision repair (NER), base excision repair (BER), and mismatch repair (MMR) [17, 31–34] (Box 2). Multiple DNA repair proteins, belonging to different pathways, cooperate to remove specific types of DNA damage. As discussed in Box 2, interstrand crosslinks (ICLs) activate Fanconi anemia (FA), HR, and NER factors, while oxidative damage induces both BER and NER [35]. Moreover, different repair systems can reciprocally influence each other, as for instance, defects in FA pathway could impair Alt (alternative)-NHEJ by favoring C (canonical)-NHEJ [36]. Importantly, a different type of DNA damage may arise consequently to the intervention of a repair system, as revealed by single-strand breaks (SSBs) induced by BER glycosylases, or by unrepaired SSBs colliding with the replication fork, and finally leading to DSBs [37].

The interplay between different repair pathways is further supported by the synthetic lethal interaction observed between BER and HR defects, suggesting new therapeutic strategies, as the use of Poly(ADP-ribose)polymerase inhibitors (PARPis) to target HR-deficient tumors. In the presence of DNA breaks, PARP1 catalyzes its own ADP-ribosylation and the modification of other proteins, thus modifying chromatin structure at the sites of damage. This modification induces the recruitment of additional repair factors, including the BER scaffold protein XRCC1 (X-ray repair cross-complementing protein 1) [38]. The aberrant activity of PARP1 can impair transcriptional recovery during repair by excessively recruiting the ubiquitin protease USP3, which reduces ubiquitinated histone levels [39]. Moreover, PARP1 activity is regulated by ubiquitination-mediated degradation [40].

XRCC1 itself is subject to various post-translational modifications, such as poly-ADP-ribosylation, phosphorylation, and ubiquitination as well [41, 42]. Sensitivity to PARPis has also been observed in non-canonical HR-defective cells, which often show impaired responses to diverse DNA-damaging agents [43]. These phenotypes are linked to altered DNA damage bypass mechanisms that depend on mono-ubiquitinated PCNA (Proliferating cell nuclear antigen) [44]. This evidence highlights the role of oxidative DNA lesions processing in influencing the sensitivity of both canonical and non-canonical HR-deficient cells to PARPis [43] and supports the importance of the cooperation among different repair pathways to maintain genome stability.

A severe risk of genomic instability may derive from chromosomal breakage and rearrangements occurring during the resolution of HR intermediates, specifically double Holliday junctions (dHJs). This is exemplified by the elevated crossover events seen in cells from Bloom syndrome (BS) patients [45]. As well as FA, ataxia telangiectasia (A-T) and Nijmegen breakage syndrome (NBS), BS is indeed classified as a severe chromosome instability disorder, characterized by predisposition to cancer [46]. Mutations in the RecQ helicase Bloom (BLM), a component of the BTR complex with Topoisomerase IIIα (TOP3A), RMI1 (RecQ-mediated genome instability protein 1), and RMI2, impair the dissolution of dHJs into non-crossover products, leading to increased crossover events. The regulation of BLM’s anti-crossover activity has been extensively studied. Phosphorylation events trigger protein-protein interactions and create a positive feedback loop aimed at suppressing crossover recombination and limiting genomic instability [45]. Interestingly, ubiquitination also plays a crucial role in regulating BLM function, being necessary for its recruitment to stalled replication forks [47].

The presence of SSBs and DSBs clearly promotes post-translational modifications such as phosphorylation and ubiquitination. Ubiquitination of chromatin and associated proteins at damage sites coordinates the DDR by signaling the recruitment of repair factors or promoting proteasomal degradation to regulate repair factor levels [48]. Ubiquitination also modulates the activity of the repair mechanism. For example, the FA pathway is responsible for repairing DNA ICLs that stall replication forks, primarily functioning through the ubiquitination activity of the RING E3 FA core complex [49]. Overall, DDR coordinates cell cycle checkpoint activation and repair processes, displaying a crucial role to preserve genome stability, as sustained by cancer predispositions in DDR-defective syndromes [46] and by the highly frequent mutations in DDR genes reported in multiple cancers [50, 51]. A fine-tuned regulation of signaling recruitment and activation of specific factors or pathways is indispensable for a properly functional DDR.

BOX 2 DNA Damage Response (DDR) and repair MMR includes factors such as MutS (mutator S) α and β (MutS protein homolog MSH2-MSH6 and MSH2-MSH3), recognizing mispairs induced by DNA polymerases that escape proofreading [208] and insertion/deletion loops (IDLs), resulting from errors during replication or recombination [31, 208]. After recognition of a DNA lesion, the excision of the patch containing it occurs by the incision activity of the MutL (MLH, mutator L homolog) complex (MutLα (MLH1-PMS2, Postmeiotic segregation increased 2 complex), MutLβ (MLH1-PMS1), or MutLγ (MLH1-MLH3)). The strand is finally corrected by excision, DNA synthesis, and ligation, involving PCNA (Proliferating cell nuclear antigen), RPA (replication protein A), RFC (replication factor C), Exonuclease I (Exo I), DNA polymerases δ and ε, Flap endonuclease 1 (FEN1), and additional factors [208, 209].

The stabilization of MMR proteins by post-translational modifications and proteasomal degradation [62, 210] underlines the importance of ubiquitination events in the regulation of repair proteins’ function.

Crosslinks and helix-distorting DNA adducts, such as 6-4 photoproducts (6-4PPs) and pyrimidine dimers (CPDs) induced by UV rays, activate NER, which is divided in GG (global genome) -NER and TCR (transcription-coupled) -NER, depending on whether the damage occurs in a transcriptionally active or inactive domain [31, 211]. In GG-NER, DNA lesions are detected by the Xeroderma pigmentosum factor C (XPC)—hRad23B/A protein complex and by the DDB1 (DNA damage-binding protein 1)—DDB2 (or XPE) complex, while the arrested transcription apparatus signals the DNA damage in TC-NER, with the involvement of Cockayne syndrome (CS) proteins CSA and CSB [31, 71, 211]. Their participation in DNA damage-induced ubiquitination events triggers the subsequent repair process, ensuring the removal of RNA polymerase II (RNAPII) for degradation [67, 69, 212]. The transcription factor TFIIH (transcription factor II H) acts through the helicase function of its XPB and XPD subunits. The excision of a 30 nucleotides-long DNA fragment containing the lesion occurs after the incision step performed by XPF and XPG nucleases. The DNA Polδ/ε, in cooperation with PCNA and RFC, synthesize a new DNA fragment. Finally, Ligasi I, or Ligase III with the help of the BER scaffold protein XRCC1 (X-ray repair cross-complementing protein 1), operates the sealing step [31, 71, 211]. Interestingly, ubiquitination also regulates the late stages of TC-NER, when CSB is degraded by the activity of the CSA, including E3 complex [72].

Notably, the NER pathway is also activated by the oxidatively induced bulky lesions cyclopurines, which represent an exceptional type of DNA damage, considering the common view that oxidative DNA lesions are mostly repaired by the BER [35]. Indeed, NER factors cooperate with BER proteins in response to oxidative stress. For instance, CSB interacts with multiple BER factors to improve their recruitment or stimulating their function, both in the nuclear and in the mitochondrial compartment [213].

In BER, a DNA glycosylase excises the damaged base leaving an AP (apurinic/apyrimidinic) site and an endonuclease creates an SSB (single-strand break). In the short-patch sub-pathway, one nucleotide is replaced by Polβ and the gap is sealed by XRCC1-Ligase IIIα. During the long-patch BER, Polδ/ε synthesize 2–10 nucleotides and FEN1 removes the 5’ flap DNA, whereas the ligation is performed by the PCNA-Ligase I complex [214, 215].

During the repair process, new types of DNA damage may also arise. For example, oxidation of DNA bases caused by reactive oxygen species (ROS) activates mono- or bi-functional glycosylases involved in BER [214, 215]. Among bifunctional glycosylases, 8-oxoguanine DNA glycosylase-1 (OGG1) specifically responds to guanine oxidation by cleaving both the glycosidic bond and the phosphodiester backbone [214, 216], with its activity regulated by ubiquitination [77]. In the case of mono-functional glycosylases like MutY DNA glycosylase (MUTYH), the process involves recruiting an AP endonuclease, such as APE1, which then cleaves the phosphodiester bond, creating an SSB as an intermediate in BER [216].

SSBs are recognized by Poly (ADP-ribose) polymerase (PARP) 1, which ADP-ribosylates itself and other factors at the site of breaks, and recruits the scaffold protein XRCC1, involved in both BER and SSBs direct repair [38]. The BER-induced SSBs can pose risks to transcription and replication; if left unrepaired, they may collide with replication forks, leading to DSBs, which are typically repaired by the HR pathway [37].

In response to DSBs, a signaling cascade involves kinases such as ATM (ataxia telangiectasia mutated), ATR (ataxia telangiectasia and Rad3-related), mutated in A-T, and DNA-PKcs (DNA-dependent protein kinase catalytic subunit). These kinases phosphorylate multiple substrates, promoting the recruitment of DDR factors, cell cycle arrest, and repair processes. The choice between HR and NHEJ is tightly regulated throughout the cell cycle [217]. HR is mainly active during late S and G2 phases, when sister chromatids or homologous sequences are available as templates for recombination. In contrast, NHEJ operates throughout the cell cycle, especially in G1, but is less accurate and more error-prone because it lacks a homologous template and may involve degradation of DNA ends [18, 218], which makes the choice of the repair mechanism significantly impacting on genome stability.

The key NHEJ factor, 53BP1 (p53 binding protein 1), was first identified as a DNA damage checkpoint protein. Alongside BRCA1 (breast cancer gene 1), a RING E3 and a well-known tumor suppressor involved in HR and often mutated in cancer [50, 219], 53BP1 plays a central role in the choice of the repair pathway following DSBs [219].

In HR, BRCA1 acts upstream of BRCA2 (breast cancer gene 2), facilitating the resection of DSB ends. BRCA2 then loads the recombinase RAD51 onto resected single-stranded DNA (ssDNA), initiating strand invasion for homology search and recombination [220]. The process of DNA end resection begins with CtBP (C terminus-binding protein)-interacting protein (CtIP) working together with the MRN complex (MRE11, Meiotic recombination 11, RAD50, and NBS1, Nijmegen breakage syndrome protein 1) to generate short ssDNA at the break ends [221]. This resection produces 3’ overhangs, allowing RAD51 to form nucleofilaments that displace RPA and invade the homologous template, forming D-loop structures for homology search [220]. Once a polymerase has extended the 3’ end of the invading strand, the ligation by DNA Ligase I yields to a heteroduplexed DNA structure. Finally, the recombination intermediates like dHJs are resolved and the correction of the DSB is complete [31, 218].

While in S phase, BRCA1 promotes the removal of 53BP1 to enable resection, in the NHEJ pathway, which does not require sequence homology, 53BP1 negatively regulates end resection during G1 [90, 91, 218]. Differently from HR, NHEJ acts in an error-prone manner. Ku heterodimer (Ku70-Ku80) initiates NHEJ by binding to the free DNA ends of the DSB and recruiting DNA-PKcs, with which it forms DNA-PK complex [222]. DNA-PKcs is recruited with the endonuclease Artemis, undergoes autophosphorylation and activates the nuclease [223]. Activated by DNA binding, DNA-PKcs additionally phosphorylates Ku proteins, Ligase IV and its cofactor XRCC4 (X-ray repair cross-complementing protein 4) [222, 223]. The final ligation of DNA ends by Ligase IV completes the C (canonical)-NHEJ mechanism [222, 224].

Recent evidence indicates the Alt (alternative)-NHEJ, also named microhomology-mediated end joining (MMEJ), as an error-prone back-up pathway when C (canonical)-NHEJ or HR fail to repair [225]. This pathway requires a 2–20 nucleotides homology sequence to start [226]. PARP1 recognizes DNA breaks and activates MRN/CtIP for DNA end resection, which causes the exposition of microhomology sequence [227]. DNA ends are aligned via the microhomology sequences, and non-homologous 3′ tails are degraded by XPF. PolQ fills the gaps [228] prior to ligation by Ligase III/XRCC1 (X-ray repair cross-complementing protein 1) or Ligase I [229].

Alt-NHEJ relates to the other DSBs repair pathways in a mutual regulation and competition manner: PARP1 and Ku proteins compete for DNA breaks sensing [227, 230]; PolQ interacts with RAD51, removing RPA from resected DSBs [228]; ligation by Alt-NHEJ dependent Ligase III opposes C-NHEJ Ligase IV activity [229]. On the other hand, functional HR or C-NHEJ suppresses Alt-NHEJ [231]. These findings indicate that multiple mechanisms cooperate to safeguard genomic stability by counteracting the harmful effects of aberrant Alt-NHEJ activation [225, 226]. As well as for other DDR mechanisms, the proper coordination of DSB repair pathway choice is essential for maintaining genome stability [232].

Additionally, the removal of ICLs, highly cytotoxic covalent links between the double helix, is triggered by FA pathway in cooperation with NER and HR factors [31]. The FA protein complex, formed by FANCM and FAAP24, recruits a large multi-subunit ubiquitin ligase, termed the core complex, to DNA lesions. This structure is composed of FANCA-FANCG, FANCC-FANCE-FANCF and FANCB-FANCL-FAAP100 [233]. The core complex mono-ubiquitinates FANCD2 and FANCI, which then localize to DNA repair foci together with FANCD1, FANCJ, and FANCN [233, 234]. ICLs final repair occurs through the collaborative activity of FA, NER, and HR factors [31].

## Role of ubiquitination in DNA damage signaling and repair

Ubiquitination coordinates DDR and repair at multiple steps [1, 52–54], from histone modification for chromatin remodeling-dependent recruitment of repair factors, to repair protein ubiquitination for degradation and regulation of their activity. Chromatin remodeling involves a variety of histone modifications, including ubiquitination, which collectively facilitate DDR activation and the repair mechanisms [55–57]. Depending on the specific E3 catalyzing H2A modification, different DNA repair pathways may be activated [58, 59]. The specific lysine residue on histone H2A also influences the progression of the response to DNA damage [55]. In addition, H2B ubiquitination is crucial for damage checkpoint activation and initiation of repair [59], while ubiquitination of H3 and H4 plays an important role in response to UV-induced DNA damage[60].

Ubiquitination significantly influences the levels of DDR factors by targeting them for proteasomal degradation. For instance, regulation of p53 by the RING E3 MDM2 through ubiquitination ensures proper turnover of key DNA repair proteins, maintaining a balance between activation and resolution of repair pathways. Additionally, in the presence of mismatches, the levels of hMut (mutator) Sα protein represent a limiting factor for MMR activity. As for other repair proteins [61], the stability of hMutSα is regulated by a RING E3-mediated ubiquitination event [62]. In response to UV rays-induced bulking lesions, as well as to oxidative damage-causing helix-distorting lesions, the NER is activated. At the early stages, the sensor factor XPC (Xeroderma pigmentosum C) is ubiquitinated by the DDB1 (DNA damage-binding protein 1) - CRL4 (Cul (Cullin) 4A-RING E3 ligase) complex, which enhances its affinity binding to the damaged DNA site [63]. The role of NER proteins in oxidative damage response is quite well described [35, 64], particularly with respect to TC TCR (transcription-coupled)-NER CS proteins, CSA, and CSB [65], which are directly involved in ubiquitin/proteasome degradation events, finally regulating transcription, cell division [66], and DNA repair. CSA is indeed part of the CRL4 complex (CSA/DDB1/Cul4A), while CSB harbors a ubiquitin-binding domain [67]. Their role in recognizing the stalled RNA polymerase II (RNAPII) and in promoting its ubiquitination-mediated proteasomal degradation, as well as that of the cyclic AMP-dependent transcription factor ATF3 (Activating transcription factor 3), is crucial for the proper recruitment and activation of other NER factors [67, 68]. Therefore, both DDB2 (or XPE) and CSA proteins have been shown to associate with DDB1 and CUL4A, in complexes displaying E3 activity [69]. The recruitment of XPC during GG (global genome)-NER relies on chromatin remodeling ubiquitination events mediated by the CUL4/DDB1/ROC1 (regulator of cullins 1) E3 complex, including the ubiquitination of histones H3 and H4 [60, 70]. DDB1 poly-ubiquitinates XPC and XPE, to increase XPC affinity to DNA or to induce the degradation of XPE [63, 71]. Finally, CSB is targeted for degradation by the E3 activity of CSA/DDB1/CUL4A complex [72]. Interestingly, CSA and CSB are also reported to interact with the tumor suppressor p53, and to regulate its ubiquitination by MDM2 [73]. Given the involvement of the tumor suppressor in the regulation of the aging process [26, 74], a defect in the ubiquitination activity of MDM2, mediated by dysfunctional CSA and CSB [73], might potentially contribute to the development of the premature aging features observed in CS patients [26, 67, 73, 74].

BER activation in response to oxidative stress is regulated by ubiquitination, involving both RING- and HECT-type E3s, which target multiple DNA glycosylases, finally influencing their activity. As an example, the RING E3 tripartite motif 26 (TRIM26) regulates glycosylase endonuclease III homolog (NTH1) protein levels [75]. Moreover, in cooperation with a HECT-type E3, TRIM26 mediates the ubiquitination and degradation of the DNA glycosylase endonuclease VIII-like protein 1 (NEIL1) [76]. Additionally, the main BER glycosylase OGG1 (8-oxoguanine DNA glycosylase-1) is ubiquitinated for proteasomal degradation by an HECT E3 [77]. The function of the main DNA breaks sensor, PARP1, determining the recruitment of the BER factor XRCC1 to the sites of the lesions, is critically regulated by post-translational modifications. In particular, ubiquitination events target PARP1 for proteasomal degradation [40]. In a similar manner, XRCC1 undergoes multiple modifications, such as poly-ADP-ribosylation and phosphorylation. Importantly, ubiquitination regulates its function and stability, consequently influencing repair efficiency [41, 42].

When DDR needs to be activated, as by the most deleterious form of damage, DSBs, cell cycle checkpoint activation is coordinated by cyclin-dependent kinases (CDKs), which in complex with their cyclin partners, phosphorylate target proteins involved in DDR and repair, finally facilitating DNA resection [78]. CDKs activity is firstly kept low in the G1 phase, to progressively increase during the cell cycle [79]. The availability of repair factors belonging to one or another pathway is also regulated during cell cycle, contributing to affect their competition, finally aimed at activating a specific repair mechanism. Further elucidating the role of RING-type E3s in DDR, RNF (RING finger protein) 8 and RNF168 are mainly involved in early stages of DSBs response being, together with ATM (ataxia telangiectasia mutated), MDC1 (mediator of DNA damage checkpoint 1) and MRN (MRE11, Meiotic recombination 11, RAD50, and NBS1, Nijmegen breakage syndrome protein 1) complex, among the earliest factors found in DNA damage-induced foci [80]. Pointing to their involvement in initiating DSBs-dependent DDR, after the key event of histone H2A variant X (H2AX) phosphorylation (γ-H2AX) [81], RNF8 ubiquitinates γ-H2AX and H2A [82–84], as well as histone H1, thus amplifying ubiquitin-mediated signaling [85]. RNF168 is consequently recruited and further propagates histone ubiquitination [82–84]. RNF8 and RNF168 form K63-linked ubiquitin chains, which convert the ubiquitinated targets into recruitment platforms for downstream factors. RNF8 and RNF168 sequentially act with E2 ubiquitin-conjugating enzyme Ubc13 to generate histone K63-linked ubiquitin chains [84, 86, 87]. RNF8 induces histone ubiquitination and favors the recruitment of repair proteins [82]. It targets E2 Ubc13 to DSBs sites and stimulates poly-ubiquitination through modulating the conformation of ubiquitin linked to Ubc13 [87].

By setting a recruitment platform for DSBs repair factors, RNF8 and RNF168 influence the repair pathway choice in response to DSBs. Chromatin-targeted RNF168 rescues 53BP1 (p53 binding protein 1) recruitment for NHEJ [59], but not BRCA1 (breast cancer gene 1) for HR [87]. Moreover, RNF8 regulates the abundance of NHEJ factors at the sites of damage. In particular, it targets Ku80 for degradation via the formation of K48-linked ubiquitin chains. The depletion of RNF8 affects NHEJ efficiency, causing the prolonged retention of Ku80 at damaged DNA [88]. Additionally, RNF8 and RNF168 play an important role at the latest stages of DSB repair, being the E3s that trigger BLM recruitment to sites of replication fork stalling through its ubiquitination, thus impeding excessive HR and protecting from genome instability [47].

As in BLM patients’ cells, defects in the FA pathway are related to sensitivity to DNA damage and repair impairment. This mechanism interestingly, involves ubiquitination for proper functioning. The RING-type E3 FANCL mono-ubiquitinates the FANCI-FANCD2 (ID2) complex to trigger ICLs removal [49] and favors the recruitment of HR factors to finalize replication after repair [49, 89].

Ubiquitination is evidently and strictly involved in the regulation of the DSBs repair pathway choice. For example, the DDB1-CUL4 E3 facilitates histone mono-ubiquitination to promote DNA end resection and HR [90, 91]. Ubiquitination of H2A by BRCA1/BARD1 (BRCA1-associated RING domain 1) promotes HR [58] while ubiquitination by RNF168 favors NHEJ [59]. RNF138 also favors end resection and recombination by regulating Ku70-Ku80 proteins' displacement from the DSBs site, thus impairing NHEJ activity [92].

The tumor suppressor protein BRCA1 promotes DNA resection in HR, interacting with CtIP and by opposing to 53BP1 [93, 94]. In addition, it aids RAD51 loading through interaction with PALB2-BRCA2 [95, 96]. The RING-type E3s BRCA1 and BARD1 act in a complex that regulates histone (H2A and H2AX) ubiquitination and DNA repair factors recruitment on the sites of lesions [52, 87, 95, 97]. Its E3 activity involves various factors, including RNAPII, TFIIE (*Transcription factor II E*), NPM1 (nucleophosmin 1), CtIP (CtBP (C terminus-binding protein)-interacting protein), gamma-tubulin, ER-α (estrogen receptor alpha), and claspin [98]. Finally, in the context of the competition and mutual regulation of HR and NHEJ factors, BRCA1 ligase activity antagonizes 53BP1 in the DSBs repair pathway choice by controlling end resection [58]. Chromatin and chromatin-associated proteins’ ubiquitination is finally essential for both the recruitment of DSBs repair factors and the regulation of their function. Among the RING-type E3s involved in the DSBs response, RNF4 plays a key role by regulating the turnover of the checkpoint mediator MDC1, and of RPA (Replication protein A) at DNA damage sites. In particular, RNF4-depleted cells are reported to fail the effective replacement of RPA by BRCA2 and RAD51 on resected DNA, a critical HR step [99].

Given the involvement of ubiquitination in the regulation of DDR and repair processes, research has focused on the contributions of the E3s, which enable the substrate specificity, and are categorized into distinct families. [100]. Scientific reports mainly deal with the contribution of RING-type E3 ligases, whereas the role of HECT-type E3s remains less well understood and requires further investigation.

## The HECT E3 family

**Fig. 1:**
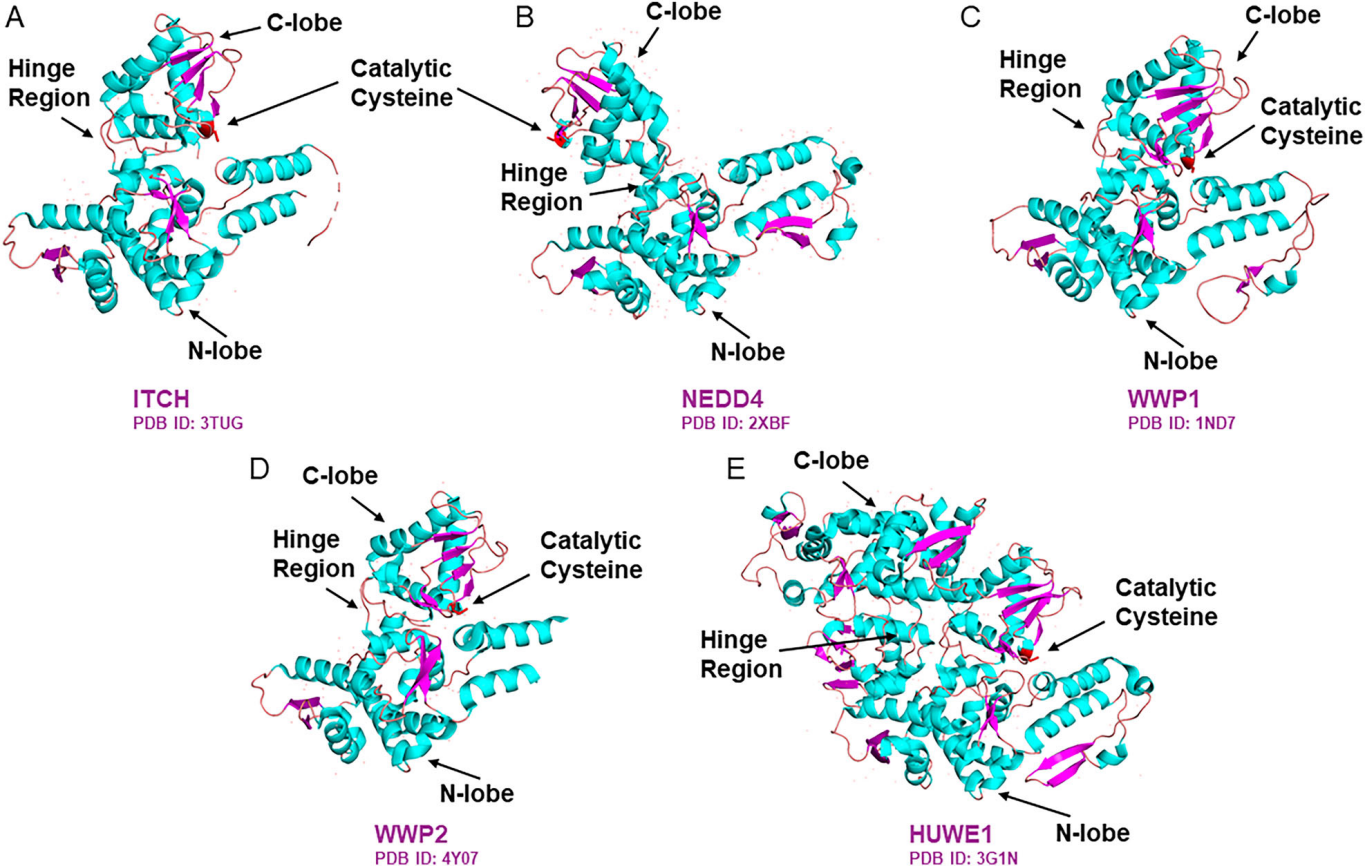
Structural domain organization of the HECT E3 family members. Structural organization of the N-lobe, the C-lobe and the hinge region (indicated with arrows) of the HECT E3s **A** ITCH, **B** NEDD4, **C** WWP1, **D** WWP2, and **E** HUWE1. The N-lobe is responsible for interacting with the E2-Ub complex, while the catalytic cysteine (in red), which forms the thioester intermediate, is located within the C-lobe. The hinge region provides the flexibility for conformational changes necessary for the catalytic function.

The HECT E3 family consists of 28 members that are characterized by the presence of a 350 amino acid-long C-terminal catalytic domain, namely the HECT domain [16]. Unlike RING-type E3s, which directly transfer the ubiquitin molecules from the E2 to the target protein [101], HECT E3s form a covalent thioester intermediate with ubiquitin prior to transferring the ubiquitin molecule to the substrate. The HECT domain itself is structurally divided into two lobes, the N-terminal lobe (N-lobe) and the C-terminal lobe (C-lobe), which are connected by a flexible hinge region, crucial for the catalytic function (Fig. 1). The N-lobe interacts with the ubiquitin-bound E2, while the C-lobe harbors the catalytic cysteine responsible for the formation of the thioester intermediate [16, 102]. The flexible hinge region allows the relative repositioning of the C- and the N-lobe, to bring the catalytic cysteine in proximity to the E2 and the substrate. The structural model from multiple HECT E3s, such as NEDD4 (Neural precursor cell expressed developmentally downregulated 4) (PDB: 2XBF) [103], ITCH (Itchy E3 ubiquitin protein ligase) (PDB: 3TUG) [104], WWP1 (WW domain-containing E3 ubiquitin ligase 1) (PDB: 1ND7) [105], WWP2 (PDB: 4Y07) [106], and HUWE1 (HECT, UBA and WWE domain containing E3 ubiquitin protein ligase 1) (PDB: 3G1N) [107], reveals such conformational flexibility (Fig. 1).

HECT E3s-dependent catalytic mechanism and conformational dynamics offer interesting possibilities to the development of selective inhibitors, targeting the catalytic cysteine or disrupting the E2-E3 interaction. Nevertheless, specificity remains a significant hurdle due to the conserved nature of the catalytic domain across HECT ligases. Accordingly, a small molecule, heclin (HECT ligase inhibitor), was found to broadly inhibit HECT E3s by inducing oxidation of the active-site cysteine, without blocking E2 binding [108]. Recent advances have identified small-molecule inhibitors of HECT family members, including NEDD4, WWP1-2, SMURF (SMAD ubiquitination regulatory factor) 1–2, and HUWE1, targeting allosteric sites and exploiting the conformational dynamics of the hinge region. Bicyclic peptides selectively target SMURF2, NEDD4, WWP1, and HUWE1, inhibiting their activity in vitro [108–110]. Interestingly, ITCH-selective inhibitors that interfere with the intramolecular domain arrangement necessary for ubiquitin transfer were developed [111]. These inhibitors exhibit promising specificity by stabilizing an inactive conformation of the HECT domain, effectively preventing substrate ubiquitination without affecting other ubiquitin ligase families.

## Involvement of HECT-type E3s in DNA damage response

**Fig. 2:**
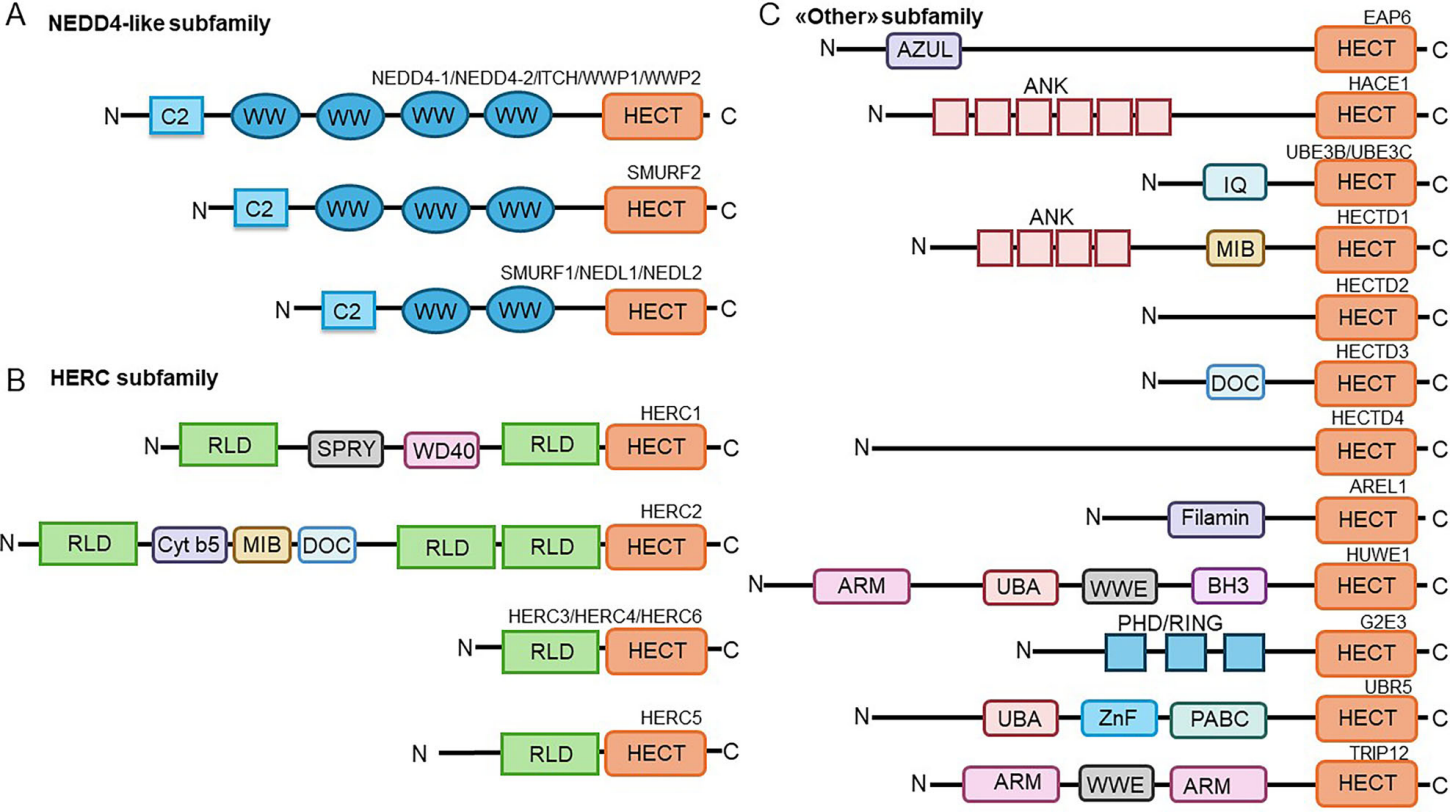
Classification of the HECT E3 ubiquitin ligase family. All members of the HECT E3 family are defined by the presence of a C-terminal HECT domain, which acts as the catalytic domain. The HECT E3 family is composed of three subfamilies. **A** The members of the NEDD4 subfamily are characterized by an N-terminal C2 domain and several WW domains. **B** The HERC subfamily includes members characterized by one or more RLD domains. **C** The “Other” HECT E3 subfamily is identified by the lack of both RLD and WW domains.

Depending on the presence of structural domains, which are responsible for the recruitment of the substrates, the HECT-type E3 family members are divided into three subfamilies: the NEDD4 (neural precursor cell expressed, developmentally downregulated 4)-like subfamily, characterized by the presence of WW (tryptophan-tryptophan) domains (Fig. 2A), the HERC (HECT and RCC1, regulator of chromosome condensation 1) subfamily, characterized by the presence of RLD (RCC1-like domain) domains (Fig. 2B) and the “other” HECT-type subfamily, which lacks both RLD and WW domains (Fig. 2C) [16].

The fate of the substrate depends on the type of ubiquitin linkages of the polyubiquitin chain. K48 and K11 linkages are mostly involved in targeting substrates for proteasomal degradation, whereas K29, K33, and K63 ubiquitin chains are implicated in autophagic processes [112, 113]. While the functions of homotypic chains are well-defined, the structure of heterotypic chains has more recently emerged, and their functions need to be further explored [114]. Importantly, specific ubiquitin chains (K6, K27, and K63) are reported to affect DDR and repair, influencing chromatin conformation and protein complexes formation, as well as the recruitment of repair factors [115](Fig. 1C).

HECT-type E3s contribute to tumorigenesis by ubiquitinating substrates that function as either tumor suppressors or oncogenes [15, 16, 102].

Being involved in the modification of histones and DDR factors, they are increasingly emerging as key regulators of effective DNA damage responses and genome stability. Nevertheless, our knowledge regarding their specific functions in these mechanisms is relatively limited compared to the extensively studied RING-type E3s. Filling this gap may offer great potential to reveal interesting novel targets for cancer therapy. In the following section, we describe the specific contribution of HECT-type E3s in the DDR and activation of repair pathways.

## The NEDD4-like HECT E3 subfamily

**Fig. 3:**
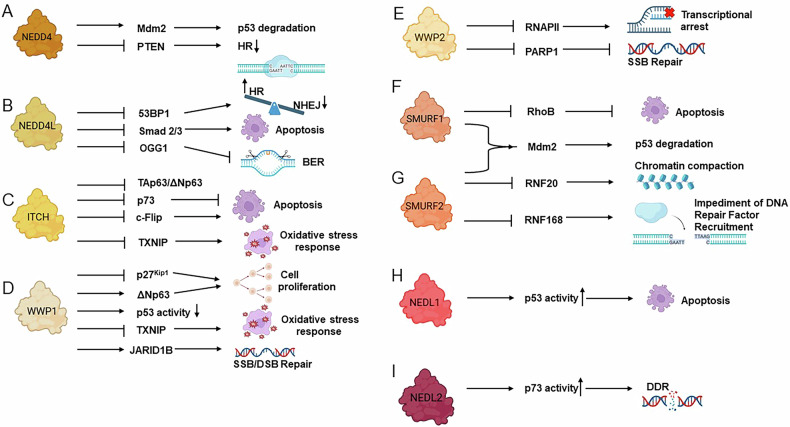
Roles of the NEDD4-like HECT E3 subfamily in the regulation of DDR. **A** NEDD4 modulates p53 activity through the ubiquitination of MDM2 and regulates HR repair by targeting PTEN. **B** NEDD4L affects the response to genotoxic stress by targeting Smad2/3 and promotes apoptosis. It regulates the balance between two DNA repair pathways (HR and NHEJ) by targeting 53BP1. NEDDL4 reduces BER efficacy by targeting OGG1. **C** ITCH controls proliferation and apoptosis by regulating p63, p73, and c-FLIP levels. **D** WWP1 modulates cell proliferation, oxidative stress response and SSBs or DSBs repair by targeting p27^Kip1, ΔNp63, TXNIP and JARID1B, while suppressing p53 activity. **E** WWP2 impairs the SSBs repair through the ubiquitination of PARP1 and the transcriptional arrest at the damage site by targeting RNAPII. **F** SMURF1 inhibits apoptosis by targeting RhoB. **G** SMURF2 modulates chromatin architecture and controls the recruitment of DNA repair factors by ubiquitinating RNF20 and RNF168. SMURF1/2 promotes p53 degradation by favoring MDM2 stabilization. **H** NEDL1 activates p53 signaling, promoting apoptosis. **I** NEDL2 regulates the DDR via modulation of p73 activity. Arrows indicate activation or promotion of the pathway, while blunt lines denote inhibition.

The members of the NEDD4-like subfamily present a forty amino acids-long conserved region containing multiple WW domains that recognize substrates proline-rich sequences (PPXY, PPLP, or PPR) (Fig. 2A). This family is composed by nine members: NEDD4 (NEDD4-1), NEDD4L (NEDD4-like, NEDD4-2), ITCH (AIP4), WWP1 (AIP5), WWP2 (AIP2), SMURF1, SMURF2, NEDL1 (HECW1, HECT, C2 and WW domain containing E3 ubiquitin protein ligase), and NEDL2 (HECW2) [116]. As detailed in the following paragraphs, NEDD4-like E3s play pivotal roles in maintaining genome stability by regulating DDR and repair mechanisms (Fig. 3).

## NEDD4

NEDD4 is involved in maintaining genome stability by regulating DDR proteins (Fig. 3A). It directly or indirectly affects the degradation of tumor suppressors, such as p53 and phosphatase and tensin homolog (PTEN), belonging to the PI3K/AKT (phosphatidyl 3-kinase/protein kinase B, PKB) signaling pathway [117]. NEDD4 physically interacts with MDM2, facilitating its stabilization through K63-poly-ubiquitination, in order to reduce p53 stability and activity [118, 119].

NEDD4 ubiquitinates PTEN, leading to its degradation and consequent protein level reduction [120].

PTEN participates in the stabilization of RAD51 and BRCA1, as well as in the regulation of the expression of ATM, Chk1 (Checkpoint kinase 1), and 53BP1. Therefore, an altered regulation of its levels by NEDD4 may result in a defective response to DSBs [119–121].

NEDD4 is also involved in RNAPII ubiquitination, thus regulating transcription-coupled repair processes. Accordingly, it favors the ubiquitination of NER proteins within the RING E3 CS complex, further contributing to DDR and protecting from genome stability [122].

## NEDD4L

NEDD4L mainly participates in DDR by regulating the stability of DNA repair proteins, thus affecting the final response to the presence of DNA damage (Fig. 3B). For instance, NEDD4L-dependent ubiquitination of 53BP1 induces its degradation. In this way, NEDD4L triggers the shifting of the repair balance toward the more error-free pathway HR [123]. NEDD4L also plays a role in controlling apoptosis during DDR. In response to genotoxic stress, it ubiquitinates SMAD2/3, thereby downregulating its protein levels and influencing TGF-β signaling pathways [124].

In addition, NEDD4L mediates the poly-ubiquitination of the glycosylase OGG1 at lysine 341, which leads to its proteasomal degradation and to the impairment of its activity during BER [77]. Impacting on BER efficiency, NEDD4L affects DNA repair, particularly under oxidative stress conditions [77, 125]. On the other hand, in the absence of NEDD4L, prolonged OGG1 stability causes an excessive excision activity, enhancing the formation of SSBs as BER intermediates [77]. Therefore, the E3-dependent regulation of OGG1 protein levels might ensure its balance to prevent genome instability. On the other hand, the expression of NEDD4L itself can be regulated by factors of other repair pathways, such as DDB2, an NER factor with transcriptional activity, shown to downregulate the expression of NEDD4L [77, 126]. Such an example of reciprocal regulation between E3s and repair enzymes underlines the importance of their cooperation for safeguarding genome stability.

## ITCH

ITCH participates in the control of transcriptional and apoptotic events, therefore significantly influencing DDR, by modulating the levels and functions of DNA repair and apoptotic factors (Fig. 3C). In this regard, ITCH catalyzes the ubiquitination of p73, a member of the tumor suppressor p53 family, for proteasomal degradation [102, 127], consequently preventing excessive pro-apoptotic signaling. Its function additionally affects the level of the other family member, p63 [128], involved in development and homeostasis processes of epithelial tissues [129]. By modulating the degradation of this substrate, ITCH affects cell proliferation, preventing tumorigenesis. Alternatively, dysregulation of such pathway may result in skin-related diseases and cancer development.

ITCH influences cell survival also by regulating the protein levels of c-FLIP (cellular FLICE-like inhibitory protein), a caspase-8 inhibitor. c-FLIP prevents caspase-mediated apoptosis by interfering with the death-inducing signaling complex (DISC). ITCH-dependent degradation of c-FLIP restores caspase-8 activity, thereby balancing apoptotic and survival responses to DNA damage [5].

Furthermore, ITCH modifies the regulator of oxidative metabolism thioredoxin-interacting protein (TXNIP) through the formation of K63-linked poly-ubiquitin chains. Notably, the subsequent degradation of TXNIP involves K48-linked ubiquitin chains, indicating a cooperative mechanism among different E3s that modulate oxidative stress responses during DDR [130].

ITCH activity is interestingly modulated by upstream DDR factors, such as ATM, which phosphorylates ITCH in response to DSBs, enhancing its catalytic activity [131].

Furthermore, AKT phosphorylates ITCH, whose nuclear localization, with the subsequent ubiquitination of the linker histone H1.2, impairs RNF8/RNF168-dependent formation of 53BP1 foci [132]. In this way, ITCH contributes to chromatin remodeling during DDR, influencing the repair pathway choice in response to DSBs, to finally favor HR.

Given its contribution to preserving genome stability, ITCH emerges as an interesting therapeutic target. Accordingly, a screening study aimed at identifying ITCH inhibitors has discovered a selective compound which blocks autophagic flux and enhances the effectiveness of chemotherapeutic agents, confirming its potential use as target in combined anticancer therapies [111].

## WWP1

WWP1 has been shown to contribute to oncogenesis by regulating tumor suppressor proteins and oncoproteins, thereby affecting cell growth, survival, and differentiation [10, 16]. Its E3 activity has been linked to multiple effects on stability, function or degradation of substrates with tumor suppressive activities, such as *p27*^Kip1^ and p53 [10, 133, 134] (Fig. 3D). For example, WWP1 catalyzes Lys-48-linked poly-ubiquitination of *p27*^Kip1^, favoring its degradation and promoting cell proliferation [135]. It has been reported to determine proteasomal degradation of the p53 family member p63 and regulating apoptosis [6]. On the other hand, it ubiquitinates the tumor suppressor p53, influencing its activity in the regulation of cell cycle arrest, apoptosis, and repair. In this regard, it has been shown that WWP1-mediated ubiquitination of p53 promotes its export to the cytosol, which results in its decreased transcriptional activities [134]. This dysregulation of p53 reduces the expression of its downstream target p21 [134].

Another WWP1-mediated nonproteolytic ubiquitination mechanism targets the p53 family member p63 isoform, ΔNp63. Catalyzing the formation of K63-based poly-ubiquitin chain on this substrate, WWP1 serves as modulator of cell proliferation [133]. In this way, the oncogenic activity of WWP1 might lead to tumorigenesis and chemoresistance. Furthermore, the downregulation of WWP1 regulates the expression of the apoptotic factors Bcl-2 (B-cell leukemia/lymphoma 2 protein) and BAX (Bcl-2-associated X protein). WWP1 also affects the expression of β-catenin, E-cadherin, MMP (Matrix metallopeptidase) -2, and MMP-9, influencing cell adhesion, migration, and invasion, which are critical for metastasis events [136, 137].

In addition, WWP1 regulates the cellular redox state and consequently, the basal levels of DNA damage through its interaction with TXNIP. Because TXNIP inhibits the disulfide reductase enzymatic activity of thioredoxin and can affect its antioxidant function, WWP1-mediated ubiquitination of TXNIP modulates redox signaling, which is important for cellular survival under oxidative stress conditions [138]. A recent study by Fierro et al. [139] reported that WWP1 is also a critical regulator of DDR, implying that its dysregulation may significantly contribute to therapy resistance, and highlighting its potential as a therapeutic target in cancer. The Authors showed that WWP1 modulates chromatin remodeling and subsequent repair of DNA breaks via modification of the histone demethylase JARID1B, which is involved in the recruitment of DNA repair factors in response to DNA damage [140–142]. WWP1 determines the formation of K63-linked poly-ubiquitin chains on JARID1B to positively regulate its stability and potentiating its demethylase activity during DDR-associated chromatin remodeling. Conversely, WWP1 silencing impairs JARID1B-dependent chromatin accessibility of HR and NHEJ factors, to finally delay the repair processes and sensitize cancer cells to chemotherapy [139].

## WWP2

WWP2 also contributes to DDR and genomic stability by regulating the levels and function of multiple factors (Fig. 3E). WWP2 catalyzes the formation of K48-linked ubiquitin chains on the large subunit of RNAPII, RPB1. During DDR, this mechanism importantly results in a transcriptional arrest at the damage site. This event, triggered by the DNA-dependent protein kinase, DNA-PK, impedes transcription to interfere with DNA repair mechanisms [143]. Conversely, defects in WWP2 significantly impact on the recruitment of DNA-PK, XRCC4, and DNA Ligase IV, thereby compromising DSBs repair by NHEJ [143].

In response to the presence of DNA breaks, the activity of PARP1 is regulated by WWP2, which performs its ubiquitination for proteasomal degradation [40]. By reducing PARP1 levels and, consequently, its PARylation activity, WWP2 prevents a putatively excessive signaling, which can be deleterious, as during isoproterenol-induced cardiac remodeling [40]. Coherently, WWP2 downregulation is associated to increased PARylation and aberrant DNA damage signaling [40].

The role of WWP1 in coordinating transcription and repair during DDR, as well as its one in modulating PARP1 activation in response to DNA breaks, points to its critical involvement in preventing genome instability.

## SMURF1 and SMURF2

SMURF1 and SMURF2 participate in DDR regulation (Fig. 3F, G), particularly downstream of the ATR (ataxia telangiectasia and Rad3-related)/Chk1 signaling pathway, being involved in the regulation of p53-dependent apoptosis in an indirect way. Their activity favors the stabilization of MDM2, which can then ubiquitinate its substrate p53 to promote its proteasomal degradation [144]. SMURF1 and SMURF2 additionally influence DDR and apoptosis in a direct way. In response to DNA damage, ATR/Chk1 activation leads to the phosphorylation of SMURF1, which then catalyzes its self-ubiquitination for degradation [145]. The reduced levels of SMURF1 lead to stabilization and accumulation of its tumor suppressor substrate RhoB, thereby promoting DNA damage-induced apoptotic events [145].

Beyond its role in the regulation of p53 during DDR (Fig. 3G) [144], SMURF2 indirectly influences damage-induced chromatin remodeling. After phosphorylation by ATM, SMURF2 ubiquitinates the RING-type E3 RNF20, which is responsible for H2B ubiquitination [146]. Blank and colleagues have reported a colocalization of SMURF2 and RNF20 at γH2AX foci at DSBs and demonstrated SMURF2-dependent degradation of RNF20, consequently affecting the mono-ubiquitination of H2B [146]. On the other hand, SMURF2 counteracts the activity of the RING-type E3 RNF168, promoting the destabilization of H2AX through ubiquitin-mediated proteasomal degradation [147]. Therefore, SMURF2 plays a dual role in the modulation of chromatin dynamics during DDR. It promotes chromatin compaction to protect DNA from damage, but also hinders the recruitment of DNA repair factors. Being activated by ATM, which is involved in DSBs-induced signaling to finally promote repair, by affecting the recruitment of repair factors, SMURF2 participates in a regulating axis that might then exert a negative feedback control of DDR and DSBs repair [148].

## NEDL1 and NEDL2

Although the roles of NEDL1 and NEDL2 in DDR are still largely unexplored, current results indicate that NEDL1 interacts with p53 to promote its transcriptional activity and subsequent apoptotic processes (Fig. 3H) [149]. This evidence suggests a potential role of this HECT E3 in the regulation of the tumor suppressor-mediated response to DNA damage.

NEDL2 has been reported to regulate DDR via its interaction with p73 (Fig. 3I). Indeed, Miyazaki and colleagues demonstrated that NEDL2 ubiquitinates p73, leading to its stabilization and to the enhancement of its transcriptional activity. In the absence of NEDL2, p73 undergoes faster decay, sustaining that NEDL2 positively regulates p73 stability [150].

The involvement of NEDL1 and NEDL2 in the modulation of the activity of p53-family members highlights them as interesting candidates for further investigations in the DDR context.

## The HERC subfamily

**Fig. 4:**
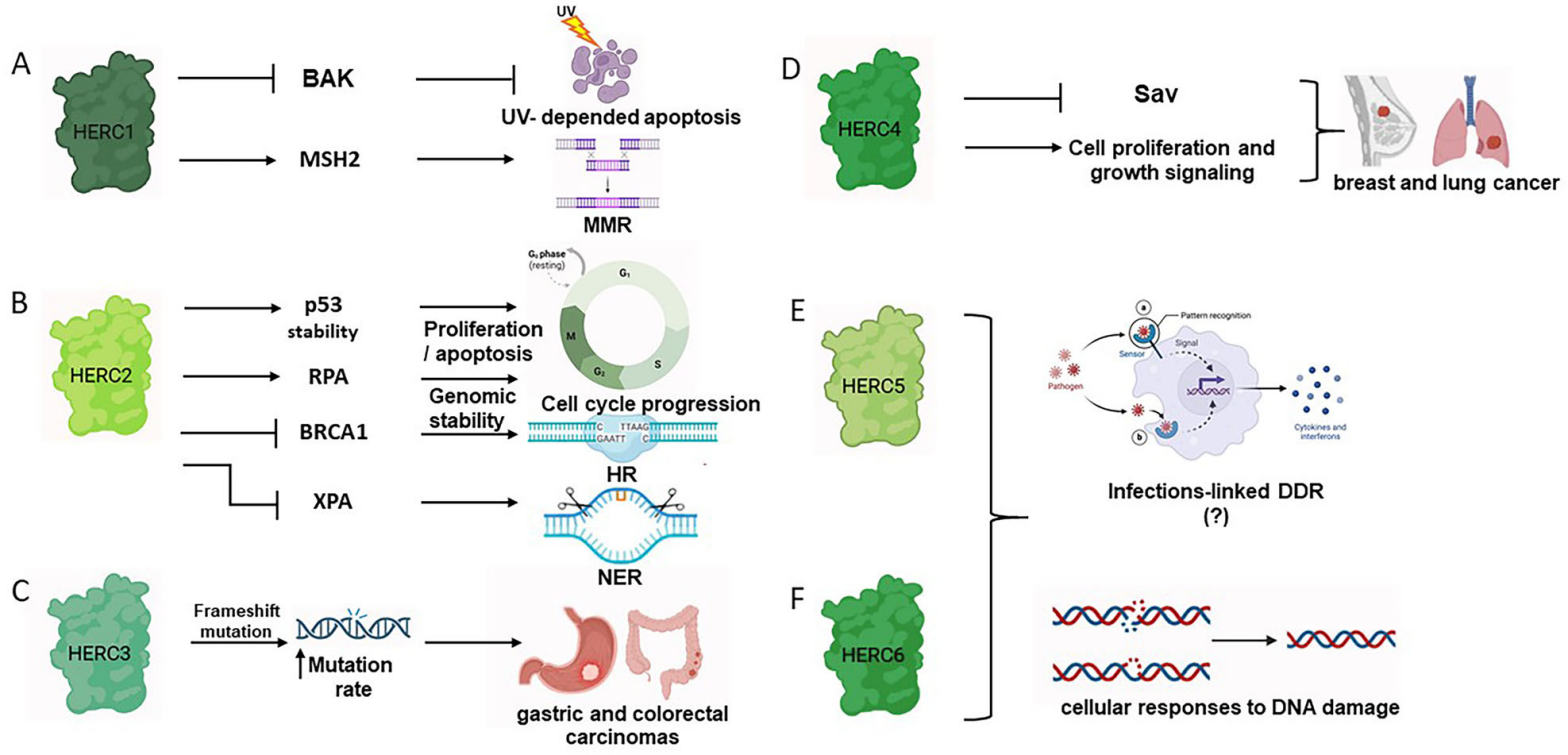
Involvement of the HERC family members in cellular processes linked to genomic stability. **A** HERC1 is shown to inhibit BAK, affecting UV damage-induced apoptosis. HERC1 also interacts with MSH2, a protein involved in MMR. **B** HERC2 stabilizes p53 through oligomerization. On the other hand, it marks p53 for degradation by MDM2 at late stages of DDR. HERC2 additionally targets both RPA and BRCA1 for degradation. **C** HERC3 is associated with frameshift mutations, increased mutation rate, leading to gastric and colorectal carcinomas. **D** HERC4 is linked to SAV1, a core component of the Hippo pathway, which regulates cell proliferation and growth signaling. Dysregulation of HERC4 is associated with breast and lung cancer. **E**, **F** HERC5 and HERC6 are involved in innate immunity, affecting signaling cascades, interferon production, and cytokine release. They might have then a still unknown role in damage response to infections affecting genome integrity. Arrows indicate activation or promotion of the pathway, while blunt lines denote inhibition.

The HERC subfamily is composed of six members, divided in large and small E3s. Whereas large HERCs (HERC1 and HERC2) present multiple RLD domains, small HERCs (HERC3-6) possess a single one [151] (Fig. 2B). Among the HERC family members, HERC1 and HERC2 are the most extensively studied for their roles in maintaining genome stability through chromatin remodeling, cell cycle regulation, and modulation of DNA repair pathways (Fig. 4).

## HERC1

HERC1 was the first member of HERC family to be identified, in a study aimed at finding human oncogenic mutations in human breast cancer cells [151]. *HERC1* gene is often mutated in metastatic triple-negative breast tumors and leukemia [152, 153]. Upon UV-induced DNA damage, in the presence of the HPV5β virus E6 protein, HERC1 impedes apoptosis by promoting the degradation of BAK (Bcl-2 homologous antagonist/killer) [7] (Fig. 4A).

Considering a direct involvement of this E3 in the regulation of DNA damage repair, it has been shown to impact on MMR, by regulating the protein levels of MSH2 (MutS Homolog 2) (Fig. 4A). Given the key role of MSH2 in the detection of replication-induced mismatches and insertion/deletion [154], the reduction of its levels significantly affects genomic stability. Diouf and colleagues [153] observed that the downregulation of HERC1 is associated with a marked reduction in MSH2 protein levels, suggesting a role in their stabilization, either directly or indirectly. Although the specific mechanism has not been elucidated, they hypothesized that HERC1 may regulate MSH2 through ubiquitination, either preventing its degradation or facilitating its proper cellular localization [153]. Given the critical role of MSH2 in maintaining genomic fidelity, HERC1 dysregulation might lead to defective MMR repair, thereby promoting mutations, genomic instability and tumorigenesis.

## HERC2

HERC2 plays a key role in modifying chromatin architecture at damage sites and helps signaling to facilitate repair. Of note, HERC2 serves as a regulator of p53 (Fig. 4B) by selectively recruiting ATM and ATR kinases, thereby modulating MDM2 and p53 stability throughout the DDR. In addition, the interaction between HERC2 and p53 is necessary for the oligomerization of this transcription factor [155]. Despite this evidence, the specific mechanism by which ATM- and ATR-dependent phosphorylation of HERC2 determines the stabilization of p53, or how HERC2 marks p53 for degradation by MDM2, has not been elucidated yet [125].

HERC2 also interacts with RPA, which plays the necessary role of protecting ssDNA from nuclease digestion during repair [156]. Under replication stress, RPA2 is phosphorylated by ATR and ubiquitinated by HERC2 for final proteasomal degradation (Fig. 4B) [157]. This mechanism is essential for the subsequent recruitment of BLM and WRN (Werner) helicases to the RPA complex, thus finally ensuring genomic stability [156, 157].

HERC2 is phosphorylated by ATM, ATR, or DNA-PK, to promote the formation of a complex with MDC1 and RNF8. The latter is subsequently induced to recruit RNF168 and the E2 enzyme UBE2N to the DSBs. In their proximity, this complex finally targets H2A and H2AX histones with K63-linked ubiquitin chains to further mark damaged DNA. These markers facilitate the recruitment of the DNA repair factors BRCA1, BARD1, RAP80 (Receptor-associated protein 80), and 53BP1, ensuring a complete damage response [125, 151]. Additionally, the E3 activity of HERC2 can target BRCA1 for degradation (Fig. 4B), counteracting the stabilizing effect of BARD1. Elevated HERC2 expression in breast tumors suggests that this regulatory mechanism may contribute to carcinogenesis [158].

HERC2 also participates in regulating NER in response to UV-induced DNA lesions. The downregulation of HERC2 results in the stabilization Xeroderma pigmentosum A factor (XPA) and enhances repair activity following cisplatin treatment, indicating that HERC2 post-translationally modulates XPA levels through ubiquitination-mediated proteasomal degradation (Fig. 4B) [151, 159]. Conversely, phosphorylation of XPA by ATM prevents its HERC2-mediated degradation, thereby maintaining a balance between repair and cell cycle progression after damage [160]. Dysregulation of this process may compromise DNA repair fidelity, increase mutation rates, and heighten cancer susceptibility [159].

## HERC3-6

Currently, our understanding of the roles played by the small HERC family members in the context of DDR and repair mechanisms remains limited. Despite this, their pathological significance and potential functional involvement in DNA repair pathways have been the subject of growing scientific interest, particularly considering their participation in various essential cellular processes such as cell growth regulation, immune responses, and maintenance of cellular homeostasis [151].

Interestingly, mutations in genes which encode for HERC family member proteins have been identified. Frameshift mutations in the HERC3 gene were found in gastric and colorectal carcinomas characterized by microsatellite instability (Fig. 4C) [161]. In invasive breast and lung cancer, HERC4 was found to be overexpressed, suggesting it as a putative marker for these types of tumors [162, 163]. Its overexpression is associated with cell proliferation and growth signaling, particularly through the regulation of SAV1 (Salvador family WW domain containing protein 1), belonging to the Hippo pathway and involved in tumor suppression (Fig. 4D) [164]. Even though the exact process determining HERC4-mediated regulation of carcinogenesis has not been deeply investigated yet, these findings suggest it has an oncogenic factor, and possible cancer therapeutic target.

HERC5 has also been proposed as a prognostic marker in patients with non-small-cell lung cancer (NSCLC) and hepatocellular carcinoma (HCC) [165, 166]. Both HERC5 and HERC6 were identified as ISG15 (Interferon-stimulated gene 15) E3s, catalyzing the conjugation of ISG15, a ubiquitin-like modifier, in response to viral infections during antiviral immune responses (Fig. 4E, F) [167, 168]. This function suggests that small HERCs could potentially play interesting roles in safeguarding genomic integrity by modulating DDR, especially when induced by infection-associated DNA damage [169, 170].

## The “Other” subfamily

**Fig. 5:**
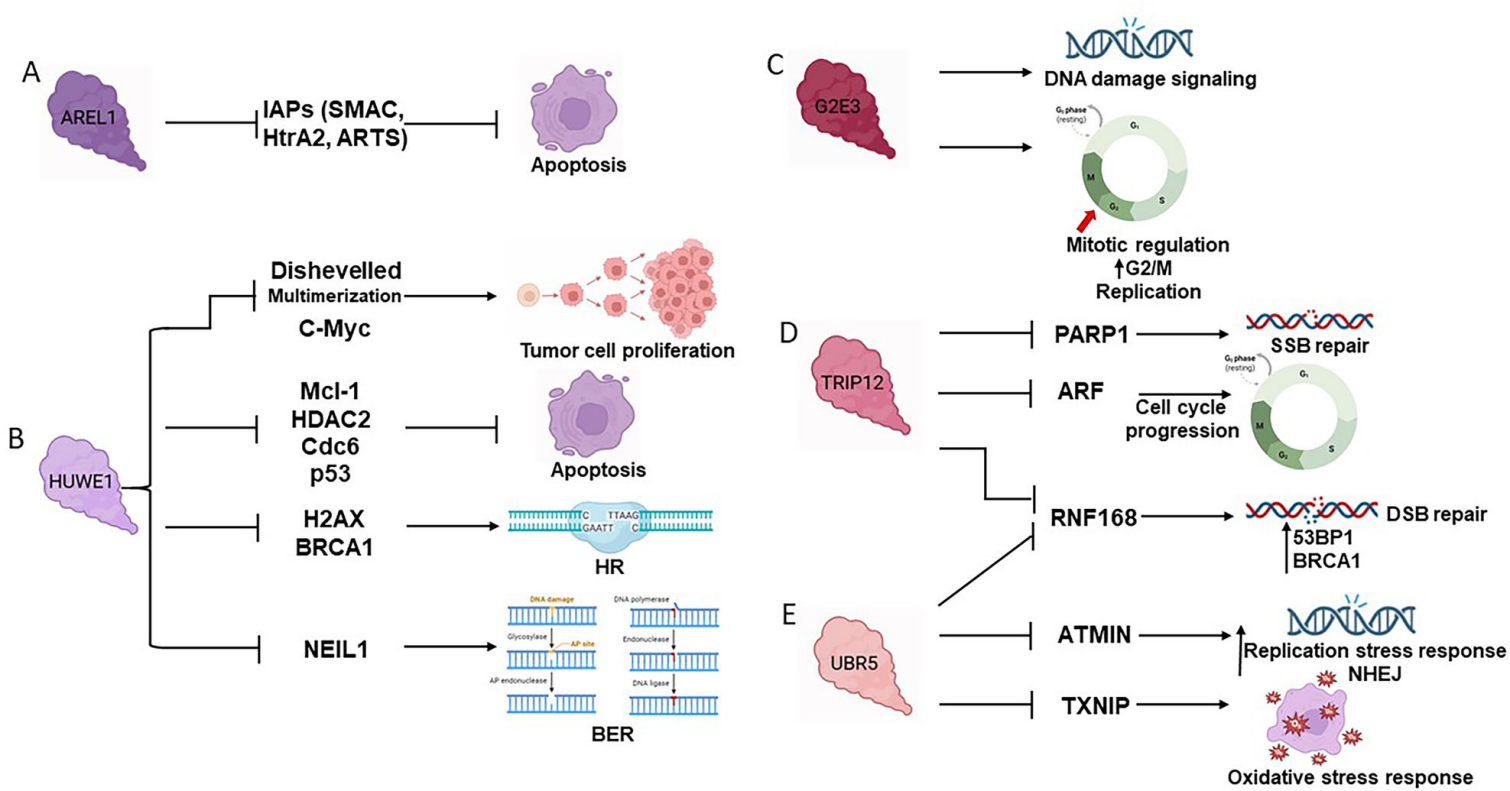
Roles of the “Other” subfamily in DDR and repair. **A** AREL1 promotes the ubiquitination and subsequent proteasomal degradation of pro-apoptotic factors, including IAP, SMAC, HtrA2, and ARTS. **B** HUWE1 targets and destabilizes key oncoproteins such as Dishevelled and C-Myc. It concurrently confers apoptotic sensitivity through its ubiquitin ligase activity on Mcl-1, HDAC2, and Cdc6. Its role in DNA integrity maintenance is highlighted by its interaction with H2AX and BRCA1 in HR repair, and its regulatory effect on NEIL1 in BER. **C** G2E3 is necessary for an efficient DDR signaling and regulation of mitosis. **D** TRIP12 significantly influences SSBs repair by modulating the stability or activity of PARP1, while its regulatory impact on ARF underscores its contribution to appropriate cell cycle transitions. Acting on RNF168, TRIP12 also regulates the recruitment of DSBs repair factors. **E** Additionally, to TRIP12, UBR5 regulates RNF168. UBR5 is involved in DDR, facilitating DNA repair upon replication stress or oxidative stress. It interacts with ATMIN and influences 53BP1 localization for initiating NHEJ. Finally, UBR5 regulates the oxidative stress response through its effect on TXNIP. Arrows indicate activation or promotion of the pathway, while blunt lines denote inhibition.

The “Other” HECT subfamily is composed of thirteen members, which are: E6AP, E6-associated protein, or UBE3A, ubiquitin protein ligase E3A; UBE3B; UBE3C; HACE1, HECT domain and ankyrin repeat containing E3 ubiquitin protein ligase 1; HECTD1, HECT domain E3 ubiquitin protein ligase 1; HECTD2; HECTD3; HECTD4; AREL1, apoptosis-resistant E3 ubiquitin protein ligase 1; HUWE1, HECT, UBA and WWE domain containing E3 ubiquitin protein ligase 1; G2E3, G2/M-phase-specific E3 ubiquitin protein ligase; UBR5, ubiquitin protein ligase E3 component N-recognin 5; and TRIP12, thyroid hormone receptor-interacting protein 12. Differently from the members of the other families, these E3s lack both WW and RLD domains (Fig. 2C). Among these thirteen enzymes, five have been primarily implicated in the regulation of apoptosis and DNA repair pathways (Fig. 5).

## AREL1

AREL1 is an anti-apoptotic E3 capable of assembling K33-, K48-, and K63-linked poly-ubiquitin chains [115, 171]. It was reported to be overexpressed in NSCLC and downregulated in multiple cancer cell lines, where it was demonstrated to favor apoptosis. AREL1 presents various ubiquitination substrates, especially among IAP (inhibitor of apoptosis) antagonists, such as SMAC (Second mitochondria-derived activator of caspases), HtrA2 (High-temperature-required protein A2), and ARTS (apoptosis-related protein in the TGF-β signaling pathway). The ubiquitination process catalyzed by AREL1 induces the degradation of such substrates, thus impairing their pro-apoptotic functions and finally aiding cancer cells in evading programmed cell death (Fig. 5A) [172].

## HUWE1

HUWE1 catalyzes the formation of K6-, K11-, K48-, and K48/K63-linked poly-ubiquitin chains on different substrates involved in the regulation of the balance between cell death and proliferation [173, 174]. For instance, HUWE1 mediates K63-linked poly-ubiquitination of Dishevelled, modulating Wnt signaling, and of c-Myc, promoting tumor proliferation (Fig. 5B) [175, 176]. Additionally, HUWE1 catalyzes the ubiquitination of the histone deacetylase HDAC2, of the tumor suppressor p53, of Mcl-1 (Myeloid cell leukemia 1), and Cdc6 (Cell division control protein 6) (Fig. 5B) [177–180]. The interactions between HUWE1 and the anti-apoptotic factor Mcl-1 occurs though a region similar to the Bcl-2 homology 3 (BH3) domain. The subsequent ubiquitination and degradation of Mcl-1 promotes DNA damage-induced apoptosis. Conversely, the downregulation of HUWE1 stabilizes Mcl-1, thereby attenuating apoptosis [178]. Following DNA damage induced by UV or alkylating agents, HUWE1 influences the levels of Cdc6, a critical component of pre-replication complexes, thereby impacting on DNA replication and repair processes [179].

HUWE1 also targets HDAC2 for ubiquitination and subsequent degradation. Zhang et al. demonstrated that loss of HUWE1 leads to HDAC2 accumulation, which impairs p53 acetylation, consequently compromising p53-dependent transcriptional activation, stabilization, and apoptotic responses upon DNA damage (Fig. 5B) [177]. In p53 wild-type cells, inactivation of HUWE1 results in p53 stabilization and activation, indicating that HUWE1 is a key regulator of p53 activity, functioning alongside the canonical MDM2 pathway and ARF (ADP-ribosylation factor)-mediated activation [180].

HUWE1 also targets H2AX [181] and BRCA1 for degradation (Fig. 5B) [182]. Affecting H2AX dynamics during DDR, it might influence the recruitment of repair factors at the sites of damage. Additionally, HUWE1-mediated proteasomal degradation of BRCA1 might affect HR response to DSBs. Moreover, in the context of BER-dependent response to oxidative damage, together with the RING-E3 TRIM26, HUWE1 targets the BER glycosylase NEIL1 for ubiquitination-dependent degradation (Fig. 5B) [76].

## G2E3

G2E3 has been identified as a mitotic regulatory protein that is specifically active during the G2/M phase and functions in response to DNA damage [183]. Studies using G2E3 knockout mice demonstrated its role in inhibiting apoptosis during early embryonic development, highlighting its importance in cell survival [184]. In terms of cellular localization, G2E3 mostly accumulates in the nucleolus but relocates to the nucleoplasm under genotoxic stress conditions, suggesting a direct involvement in DDR [183]. A comprehensive siRNA screening targeting 327 human E3s revealed G2E3 as a novel factor involved in damage response. Depletion of G2E3 led to a reduction in γH2AX levels and impaired phosphorylation of Chk1 following cisplatin treatment, indicating a compromised DNA damage signaling (Fig. 5C). Additionally, loss of G2E3 induced apoptosis and resulted in decreased proliferation of cancer cells. Treatment with the nucleoside analog gemcitabine further revealed that G2E3 depletion causes the accumulation of ssDNA, indicative of replication stress (Fig. 5C). Importantly, endogenous G2E3 levels are downregulated in cancer cells following chemotherapy exposure [185]. Collectively, these findings suggest that G2E3 participate in DDR, protecting from replication stress and genomic instability [185, 186].

## TRIP12

TRIP12 was initially identified as a binding partner of the thyroid hormone receptor [187], as well as a factor indirectly involved in p53 degradation [188]. Its expression was found to be altered in multiple diseases, underlying it as a potential therapeutic target [189]. Regarding its role in DDR, Altmeyer’s laboratory demonstrated that TRIP12 binds PARP1 via a PAR-binding domain and catalyzes its poly-ubiquitination for proteasomal degradation (Fig. 5D) [190]. By regulating PARP1 levels, TRIP12 limits the PARP1-trapping effect of PARPis at sites of DNA breaks, whereas the downregulation of TRIP12 enhances PARPi efficacy, leading to increased DNA replication stress and damage [190]. According to such evidence, TRIP12 might be a promising candidate for therapeutic strategies aimed at overcoming resistance mechanisms in BRCA-deficient tumors.

TRIP12 also acts as a tumor suppressor by modulating the ARF-p53 pathway [191, 192]. Chen and colleagues demonstrated that knocking down TRIP12 stabilizes ARF in normal human cells, resulting in ARF-dependent p53-mediated growth arrest (Fig. 5D) [191].

Additionally, Gudjonsson and collaborators revealed that TRIP12, along with UBR5, regulates the levels of RNF168 [192]. Their findings indicate that RNF168 excessively accumulates as DSBs levels increase, especially in dependence on the depletion of TRIP12 and UBR5, thus resulting in the excessive recruitment of 53BP1 and BRCA1 (Fig. 5D) [192].

Poulsen and colleagues demonstrated an interesting functional correlation between TRIP12 and HUWE1 in the ubiquitin fusion degradation pathway. Their double knockdown caused an additive stabilization of shared substrates, suggesting that these enzymes operate in parallel [193]. The functional redundancy between TRIP12 and HUWE1 suggests that they may share additional common substrates involved DDR, further supporting the importance of ubiquitin-mediated events in the maintenance of genome integrity.

## UBR5

In addition to its roles in the modulation of RNF168 levels and in histone ubiquitination for DNA damage-associated chromatin remodeling, together with TRIP12 (Fig. 5D-E) [192], UBR5 participates in the ubiquitin-proteasome system in tumors. UBR5 is indeed highly expressed in colorectal cancer, where it shows oncogenic functions [194].

Importantly, UBR5 participates in the early stages of DNA repair. The peroxisome proliferator-activated receptor-γ (PPARγ) enhances UBR5-mediated ubiquitination and subsequent degradation of the ATM-interacting protein ATMIN (Fig. 5E) [195]. Since ATMIN is essential for the proper localization of 53BP1 under replicative stress, its regulation by UBR5 influences NHEJ efficiency in response to DSBs (Fig. 5E) [195, 196].

Furthermore, UBR5 collaborates with other E3s to assemble branched ubiquitin chains. It has been reported to facilitate ITCH in forming K48/K63-branched ubiquitin chains on TXNIP, thereby targeting it for degradation (Fig. 5E) [130]. This suggests a potential indirect role of UBR5 in oxidative damage response regulation. Mechanistically, TXNIP is initially targeted with K63-linked ubiquitin chains, which may serve as a substrate-specific signal to recruit other E3s, such as UBR5 and WWP1, that catalyze K48 linkages [130, 138, 192]. While K63-linked ubiquitination of TXNIP is necessary for its recognition, it is not sufficient alone for proteasomal degradation [130], which can instead be directly induced by the k48-linked ubiquitination activity of WWP1 [138]. These findings further sustain the important role of the crosstalk among different E3s in regulating DNA repair, particularly in response to oxidative stress.

## Final remarks and future directions

In conclusion, although being less studied than the members of other E3 families, HECT E3s have emerged as regulators of DDR [197], underlying the importance of ubiquitination, as well as of the crosstalk among different E3 families, in the regulation of processes aimed at the maintenance of genome integrity. By regulating the protein levels and functions of their DDR substrates, HECT E3s orchestrate the balance between cell survival and cell death upon genotoxic stress, thus preventing tumorigenesis. Furthermore, most of them show altered expression levels in association with the onset of multiple cancers and therapeutic resistance acquirement.

Notably, proteasome inhibitors show efficacy in cancer therapies [198], confirming the important role of ubiquitin-related enzymes in multiple biological mechanisms, both in physiological and pathological contexts. Proteasomal function is regulated by ubiquitination events, mediated by proteasomal-associated HECT E3s, which alter its substrates recognition and proteolytic activity, importantly, also in response to genotoxic stress [199, 200]. Investigating the involvement of specific HECT E3s in proteasomal degradation, assembly, and function, would further uncover their role in DDR.

Despite the critical roles of HECT E3s, the mechanisms dictating substrate specificity, spatiotemporal regulation, and crosstalk with other E3s or with different post-translational modifying enzymes remain poorly explored. The interplay among members of different subfamilies, determining a variety of particularly characteristic, mixed, and branched ubiquitin chains, also offers an interesting opening to additional investigations. Structural studies and high-throughput proteomic approaches could help uncover these regulatory layers. Screenings for HECT E3 inhibitors will putatively aim at identifying molecules, able to selectively target specific ligases. By affecting specific DDR mechanisms, they have good potential to therapeutically achieve improvements for patients’ therapies. The integration of screening platforms with structural-modeling approaches will possibly help to isolate selective inhibitors, finally reducing the risk of off-target effects and associated toxicity.

The development of PROTACs (Proteolysis Targeting Chimeras) represents an innovative strategy in which the targeted degradation of proteins is accomplished by recruiting specific E3s [201, 202]. Despite such a promising approach, the big obstacle of achieving specificity remains for both inhibitor and PROTAC development, due to the significant structural similarities among various HECT E3s.

The refinement of small molecule inhibitors and PROTACs will possibly enhance specificity and help to develop more effective and combined therapies to overcome resistance. Further investigations on the multiple roles of HECT E3s will then be particularly helpful to improve cancer therapies, especially where resistance remains a persistent hurdle [203].

Targeting such enzymes may modulate DDR and DNA repair pathways in cancer and diseases characterized by genomic instability, to possibly sensitize tumors to genotoxic agents or restoring DNA repair in inherited deficiencies. For instance, targeting HERC2 might affect both BRCA1 and XPA levels, influencing PARPis- or cisplatin-resistant tumors response to therapy. Inhibitors of TRIP12 or WWP2 might positively affect the sensitivity to PARPi of HR defective tumors. Potential inhibitors of WWP1 might also be deeply investigated as therapeutic agents to modulate chromatin accessibility and DDR functionality in cancer. Overall, the combined use of HECT E3s inhibitors with PARPis, oxidative treatments or radiotherapy may help in sensitizing tumors with altered DDR or repair efficiency. Finally, targeting these enzymes, or impeding the interaction with their substrates, may allow the modulation of specific pathways, thus reducing off-target effects.

## Data Availability

Availability of data and materials is not applicable in this study.
